# Supplementary figures and images for: Derivation and Clinical Validation of a Redox-Driven Prognostic Signature for Colorectal Cancer
Source: Front Oncol. 2021 Oct 27;11:743703. doi: 10.3389/fonc.2021.743703 (PMC8578893; doi:10.3389/fonc.2021.743703)

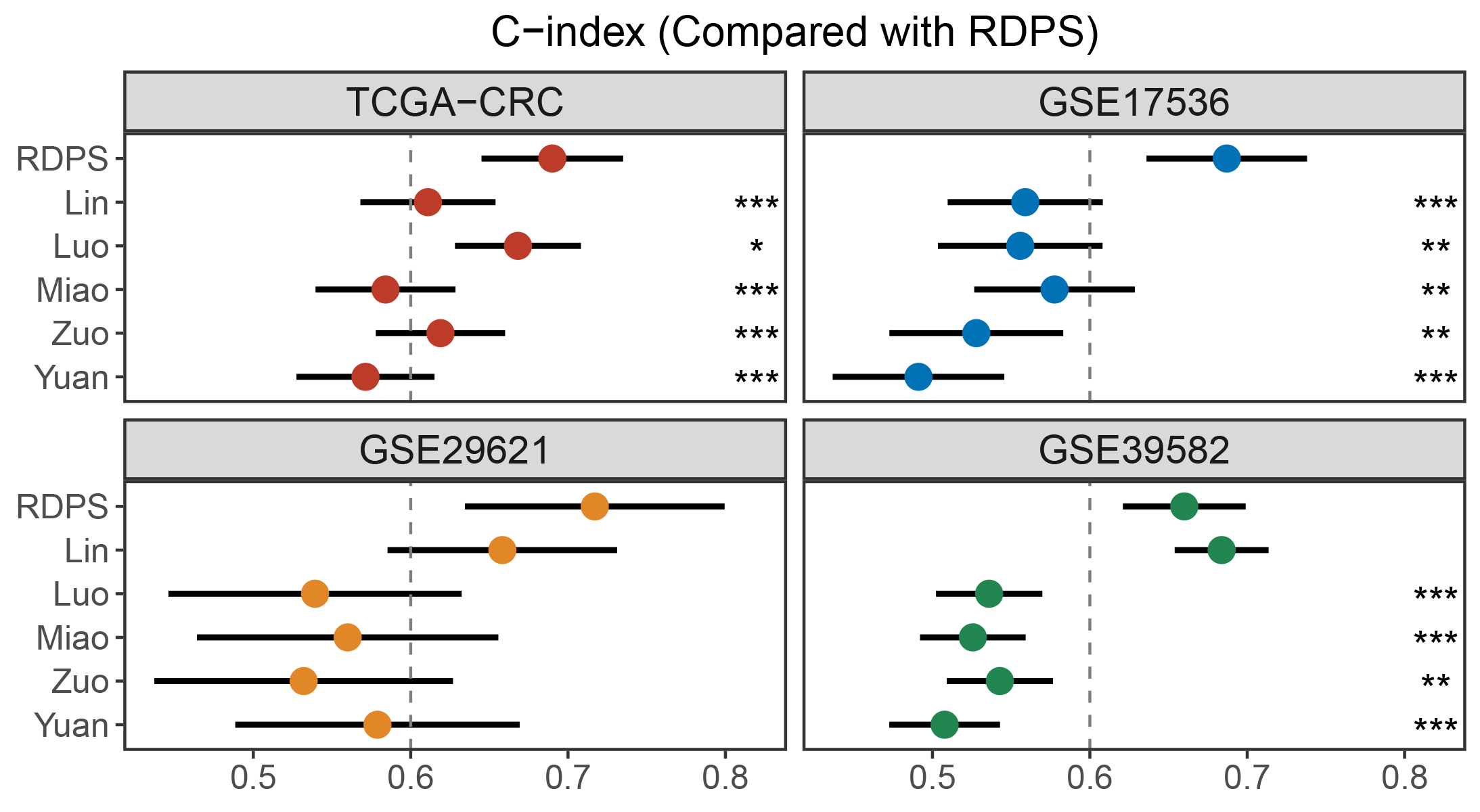

Supplement: Supplementary Figure 1 — Comparison between RDPS model and published metabolism-related signals. [file Image_1.tif]

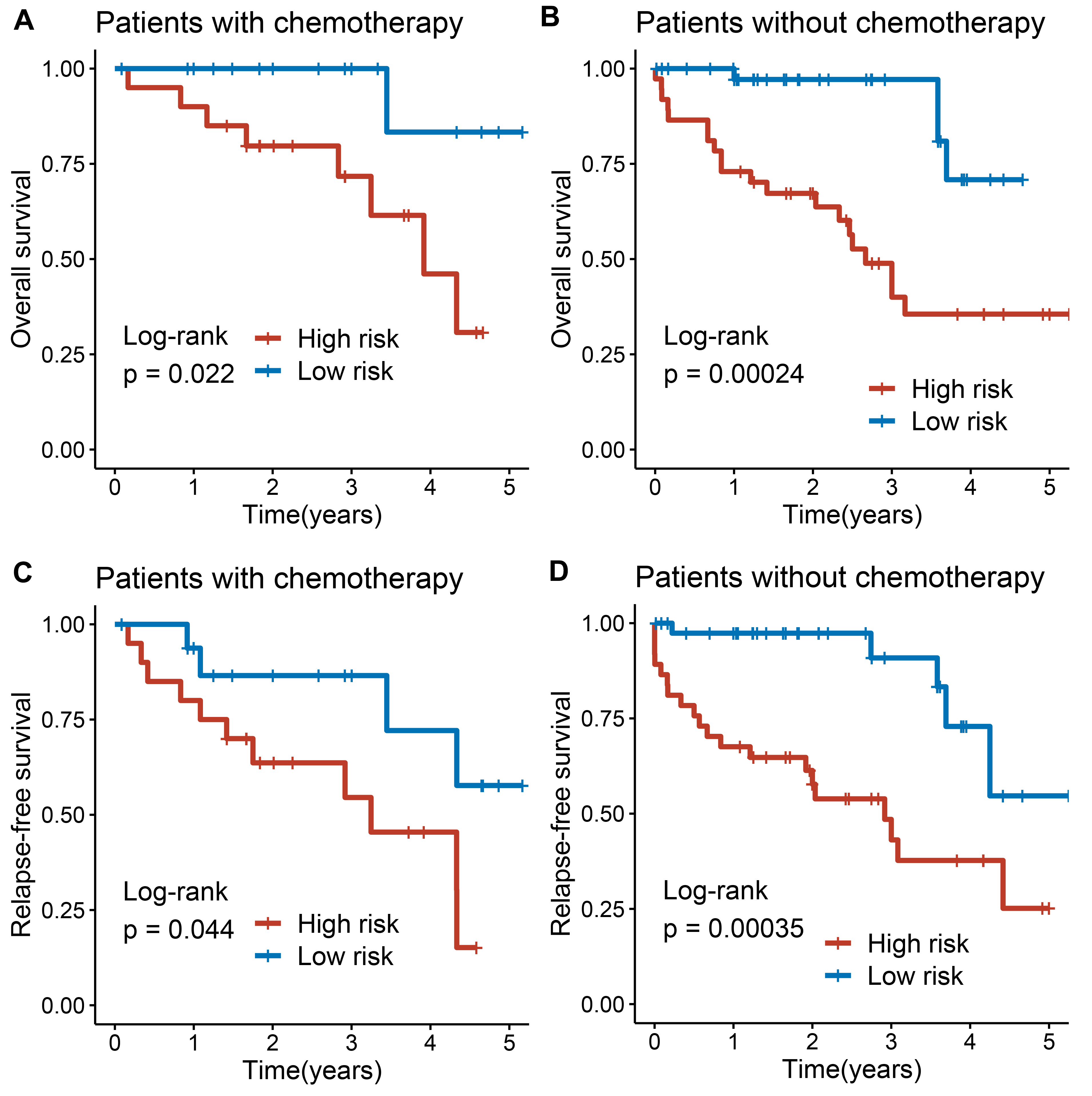

Supplement: Supplementary Figure 2 — RDPS assessment of survival in patients with and without chemotherapy. (A, B), the effect of RDPS on OS in patients receiving (A) and without (B) chemotherapy. (C, D), the effect of RDPS on RFS in patients receiving (C) and without (D) chemotherapy. [file Image_2.tif]
